# Medullary sponge kidney: in-depth phenotyping for a better understanding of functional and structural abnormalities

**DOI:** 10.1007/s40620-025-02413-3

**Published:** 2025-09-19

**Authors:** Corentin Tournebize, Aurélie De Mul, Nadia Abid, Aurélie Portefaix, Sophie Pacaud, Maxime Schleef, Laurence Derain-Dubourg, Olivier Rouviere, Sandrine Lemoine

**Affiliations:** 1 Service de Néphrologie, Dialyse, Exploration Fonctionnelle Rénale, Hôpital Edouard Herriot, Hospices Civils de Lyon, 69003 Lyon, France; 2 Centre de Références Maladies Rares Rénales, MAREGE, 69003 Lyon, France; 3 Univ-Lyon, Laboratoire CarMeN, INSERM U1060, INRAE, Université Claude Bernard Lyon 1, 69500 Bron, France; 4 Université Lyon 1, Université de Lyon, Urologie Et Chirurgie de La Transplantation, Hôpital Edouard Herriot, Hospices Civils de Lyon, 69003 Lyon, France; 5 Centre d’Investigation Clinique, Hôpital Louis Pradel, Hospices Civils de Lyon, 69500 Bron, France; 6 Service d’Imagerie Urinaire Et Vasculaire, Hôpital Edouard Herriot, Hospices Civils de Lyon, 69003 Lyon, France; 7 LabTAU, INSERM, Université Lyon 1, 69003 Lyon, France

**Keywords:** Medullary sponge kidney, Functional magnetic resonance imaging, Blood oxygen level dependent MRI

## Abstract

**Background:**

Medullary sponge kidney is an entity characterized by pre-calyceal dilatation of the renal tubules, whose pathophysiology is unknown. Tubular anomalies have been described, suggesting impaired medullary function. To better characterize these patients, tools for assessing medullary function and structure are needed. The latter can be evaluated with functional magnetic resonance imaging (fMRI), using blood-oxygen-level-dependent imaging, which quantifies tissue oxygenation, and diffusion-weighted-imaging and T1-mapping sequences which allow fibrosis assessment. The aim of this study was to deeply phenotype medullary sponge kidney patients.

**Methods:**

We carried out fMRI, measured glomerular filtration rate (mGFR) by iohexol clearance, and metabolic assessment of urolithiasis in patients with medullary sponge kidney and in healthy controls. The primary endpoint was the comparison of R2*, inversely proportional to oxygen content, measured by blood-oxygen-level-dependent MRI. Secondary endpoints included comparison of T1 and apparent diffusion coefficient, comparison of GFR between medullary sponge kidney patients and controls, and the correlations between fMRI, GFR and biological abnormalities in medullary sponge kidney.

**Results:**

Twenty patients with medullary sponge kidney were included, as well as 13 controls. We observed a higher R2* cortex-to-medulla ratio in medullary sponge kidney patients compared to controls (0.60 vs. 0.55; p = 0.04). No difference was observed for T1 and apparent diffusion coefficient cortex-to-medulla ratio. mGFR was significantly lower in medullary sponge kidney patients (90 ml/min/1.73m^2^ vs 78 ml/min/1.73m^2^; p = 0.008) although estimated GFR did not differ. Medullary cysts were visible on MRI in 60% of medullary sponge kidney patients.

**Conclusion:**

We identified impaired renal oxygenation in patients with medullary sponge kidney. We did not find evidence of kidney fibrosis in medullary sponge kidney. GFR estimation was not accurate in medullary sponge kidney patients. MRI can visualize medullary cystic appearance of medullary sponge kidney.

**Graphical abstract:**

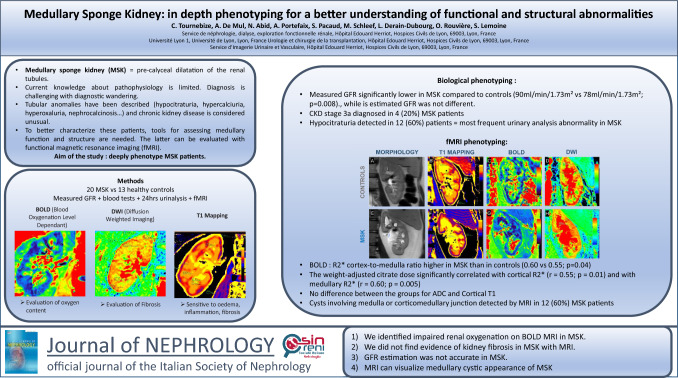

**Supplementary Information:**

The online version contains supplementary material available at 10.1007/s40620-025-02413-3.

## Introduction

Medullary sponge kidney is characterized by pre-calyceal dilatation of the renal tubules resulting in the appearance of medullary cysts and recurrent nephrolithiasis, sometimes with microcalculi in the dilated terminal tubules. Medullary sponge kidney pathophysiology remains poorly understood and its diagnosis is challenging with diagnostic wandering [1, 2]. Several tubular abnormalities favoring stone formation have been described, namely hypercalciuria, hyperoxaluria, and hypocitraturia with distal tubular acidification defect [2–4], but chronic kidney disease (CKD) is considered unusual [2].

We need a deeper phenotyping of this entity. First, considering tubular ectasia or microcalculi in the dilated terminal tubules of medullary sponge kidney, there is a need to better evaluate kidney function with measured GFR (mGFR) rather than estimated GFR (eGFR). Secondly, given the diagnostic difficulties and medullary changes, we wanted to better characterize kidney structure and medullary function. For the latter, renal functional magnetic resonance imaging (fMRI) appears to be a useful tool to evaluate cortical and medullary performance, hemodynamics, and architecture, with quantitative, non-invasive, non-irradiating, regional measurements on separate kidneys, coupled with a morphological study [5]. fMRI combines multiple pulse sequences, including blood oxygen level-dependent imaging, diffusion-weighted imaging, and magnetic resonance relaxometry. Blood-oxygen-level-dependent MRI measures tissue oxygenation based on the paramagnetic properties of deoxyhemoglobin [6]. Diffusion-weighted imaging MRI evaluates the motion of water molecules in tissues and can be used to characterize fibrosis and edema [7]. Magnetic resonance relaxometry quantifies T1 and T2 relaxation times which are characteristic of tissue composition and can quantify fibrosis, edema and inflammation [8]. Only one case of renal fMRI in medullary sponge kidney has been reported, showing a medullary hypersignal on T2-weighted imaging and medullary restriction of diffusion [9]. Further data are needed to assess fMRI relevance in this entity.

We hypothesized that mGFR and fMRI could detect and quantify the early alterations of renal medullary microstructure and function observed in medullary sponge kidney. We therefore compared different fMRI sequences and mGFR in patients with medullary sponge kidney to healthy controls and looked at associations between fMRI results and biological data. Finally, we were able to compare biological phenotypes of medullary sponge kidney patients to controls. Our aim was to deeply phenotype medullary sponge kidney patients.

## Methods

### Study design

We conducted a prospective pilot study. Participants were recruited from nephrology and urology departments of Edouard Herriot Hospital, Lyon, France, between January 2023 and December 2024. Inclusion criteria were: age ≥ 18 years, medullary sponge kidney diagnosed based on radiographic confirmation (uroscanner or intravenous urography in the delayed phase image showing the characteristic ‘papillary blush’ [10]) or ureterorenoscopic confirmation (showing the characteristic architecture of affected papillae [11]). Every diagnosis of medullary sponge kidney was made before inclusion with no doubt regarding the diagnosis. The control group consisted of unmatched adult participants (≥ 18 years) with eGFR > 60 mL/min/1.73m^2^, and no history of kidney stones. Exclusion criteria were kidney transplant, autosomal dominant polycystic kidney disease, and MRI contraindication.

### Outcomes

The primary outcome was comparison of R2* measured on blood-oxygen-level-dependent MRI between patients with medullary sponge kidney and controls. Secondary outcomes were comparison of apparent diffusion coefficient measured on diffusion-weighted imaging MRI, comparison of T1 values, comparison of GFR between medullary sponge kidney patients and controls; and correlations between fMRI parameters, GFR and tubular abnormalities in medullary sponge kidney.

### Clinical and biological characteristics

Participants’ demographic data were collected, including age, sex, weight and body-mass-index. Patients were asked about their medications. Stone analysis was recorded if available. Blood and urine biochemistry results were obtained for annual follow-up and were performed by our centralized Medical Biology Reference Laboratory (Service de biochimie et biologie moléculaire, Laboratoire de biologie médicale multisite, Hospices Civils de Lyon, France). Blood analyses included measurement of creatinine (enzymatic technique, Architect c®, Abbott Diagnostics), sodium, potassium, chloride, bicarbonate, calcium, phosphorus, parathyroid hormone, 25-hydroxyvitamin D, 1,25-dihydroxyvitamin D, magnesium, and uric acid. Participants provided a 24-h urine collection to measure creatinine, urea, sodium, potassium, calcium, citrate, oxalate, uric acid, and phosphate. Morning urine samples were collected to measure pH, specific gravity, crystalluria and cytobacteriological examination of the urine. mGFR was obtained by iohexol clearance with the following standardized protocol: a single 6 ml bolus of iohexol (300mgI/ml; Omnipaque; GE Healthcare SAS, Vélizy-Villacoublay, France) was injected intravenously. The injected dose was determined by double weighing the syringe. Blood samples were collected from the contralateral arm after 120, 180, and 240 min. Serum iohexol concentration was measured at the Medical Biology Reference Laboratory using high-performance liquid chromatography ultraviolet light detection. mGFR was calculated as mGFR = slope × dose/concentration at time 0 corrected with Bröchner–Mortensen equation and indexed to body surface area (BSA) determined by the Dubois and Dubois formula (12). GFR was also estimated by the CKD-EPI Equation (2009) [13].

Tubular creatinine secretion was estimated as the difference between measured creatinine clearance and measured GFR. Creatinine clearance was determined using the standard 24-h urine collection method, calculated as: Creatinine clearance (mL/min) = (Urinary creatinine concentration (µmol/L) × Urine volume (mL/24 h)) / (Plasma creatinine concentration (µmol/L) × 1440 min). All clearance values were indexed to body surface area (mL/min/1.73 m^2^) unless otherwise specified.

To ensure accuracy of the 24-h urine collection, participants received detailed instructions, and collection adequacy was verified by comparing total urinary creatinine excretion to expected reference values adjusted for sex and body weight. We controlled correct 24-h urine collection according to theoretical standards that we calculated using the average of the published formulas (14, 15). Urine collection was considered complete when the measured 24-h urine creatinine was within approximately 10% of the calculated theoretical value.

### MRI protocol

Participants underwent MRI using a 3.0-T MR scanner (*Ingenia* 3.0 T, Philips Healthcare, Best, Netherlands). MRI parameters are described in Supplementary Materials (Table S1)**.** Parametric maps were generated using an Intellispace Portal (v.12, Philips Healthcare) workstation. T2 sequence was used for morphological description, and MRI images were compared to the last available computed tomography (CT) scan. Total kidney and entire medullary volumes were measured using the Philips Intellispace Multi-Modality Tumor-Tracking application. Total kidney volume was normalized to BSA. Medullary volume was normalized to the total kidney volume (medullary/total kidney volume) and expressed as relative medullary volume.

Blood-oxygen-level-dependent MRI was acquired in the coronal plane using a single shot echo-planar sequence. Blood-oxygen-level-dependent signal was expressed as apparent relaxation rate R2* (s^−1^). A decrease in R2* indicates a drop in deoxyhemoglobin level and therefore better tissue oxygenation. Diffusion-weighted imaging MRI was acquired in the coronal plane using a T2-weighted echo-planar imaging sequence. Diffusion-weighted imaging signal was expressed using apparent diffusion coefficient (ADC, 10^−3^mm^2^/s). T1 mapping was acquired in the coronal plane and measurements were expressed using T1 relaxation time (ms).

Four readers (OR, SL, SP and CT), blinded to clinical or pathological information, independently and manually placed regions of interest with unfixed size in each participant and each sequence at the upper, middle, and lower pole of both kidneys, with 3 regions of interest placed on the cortex, and 3 regions of interest on the inner medulla (Supplementary Materials, Fig.S1). Region of interest selection was based on anatomical images and avoided vessels, renal sinus, and susceptibility artifacts. For each participant, cortical and medullary measurements were averaged over the four readers. The readers included one radiologist with > 20 years of experience in urinary imaging (OR), one nephrologist with 20 years of experience (SL), one clinical research radiology technician (SP), and one nephrology resident (CT).

### Statistics

Categorical and continuous variables were respectively expressed as numbers and percentages or medians and interquartile ranges (IQR). For each continuous variable, normality was assessed using the Shapiro–Wilk test and variables were compared between groups with either unpaired Student’s t-test or Mann–Whitney test, as appropriate. Paired data were compared using either paired t-test or Wilcoxon matched-pairs signed rank test, as appropriate. Categorical variables were compared between groups with Fisher’s exact test or χ^2^ test, as appropriate. Correlations were evaluated using Spearman's correlation coefficient. Correlations were tested between MRI values and the following parameters: mGFR, eGFR, 24-h urine citrate, 24-h urine oxalate, 24-h urine calcium, and weight-adjusted citrate therapy. Bilateral alpha risk of 5% was considered for significance. Missing data were not computed. Statistical analyses were performed using GraphPad Prism software (Graphpad software, San Diego, CA, USA).

## Results

### Participants’ characteristics

Twenty patients with  medullary sponge kidney and thirteen controls were included. Participants’ characteristics are described in Table 1. Two controls had incomplete 24-h urine collection. There were more female subjects in the medullary sponge kidney group (18 (90%) vs 5 (38.5%)). One control was on treatment with lercanidipine for  high blood pressure . Urine citrate was significantly lower in patients with medullary sponge kidney compared to controls (1.96 mmol/24H vs 3.68 mmol/24H; *p* = 0.01). No difference in urine calcium was observed, though 8 (40%) patients with medullary sponge kidney were on treatment with thiazides to target hypercalciuria. Hypercalciuria was detected in two patients with medullary sponge kidney, including one with dietary hypercalciuria. The median age at first kidney stone was 36 years (IQR 22–42), with pediatric onset in 4 (20%) patients. When their composition was known, stones were mostly composed of whewellite. The most frequent urinary abnormality was hypocitraturia which was detected in 12 (60%) patients, that may have been caused by hypokalemia and/or urinary colonization in 3 of these patients. Potassium citrate therapy was prescribed to 14 (70%) patients, and two patients were also being treated with another alkalinizing  agent. One patient had urinary colonization, none had urinary tract infection. Sixteen patients presented panrenal and bilateral medullary sponge kidney, three patients presented bilateral medullary sponge kidney involving not all the papillae, and one patient presented unilateral medullary sponge kidney.

**Table 1:** Participant characteristics

Variables^a^	Controls (*n* = 13)	MSK (*n* = 20)	p value
Age (years)	51 (48–59)	51 (35–56)	0.28
Female	5 (38.5)	18 (90)	0.08
Weight (kg)	73.0 (55.0–81.5)	68.5 (55.3–71.75)	0.38
BMI (kg/m^2^)	26.5 (22.9–28.8)	25.5 (20.9–28.6)	0.55
BSA (m^2^)	1.72 (1.50–1.97)	1.74 (1.60–1.81)	0.99
Systolic blood pressure (mmHg)	120 (108–123)	115 (105–125)	0.54
Diastolic blood pressure (mmHg)	71 (65–74)	66 (58–73)	0.12
Comorbidities			
Hypertension	1 (7)	0 (0)	
Diabetes	0 (0)	0 (0)	
Hyperparathyroidism	0 (0)	2 (10)	
Osteoporosis	0 (0)	1 (5)	
Plasma creatinine (mg/dL)	0.78 (0.73– 0.91)	0.80 (0.71–0.96)	0.85
eGFR (ml/min/1.73m^2^)	90.0 (84.0– 100.0)	91.00 (73.3–104.3)	0.78
mGFR (ml/min/1.73m^2^)	90.0 (81.5– 95.0)	78.0 (64.5–84.4)	0.008
mGFR < 60 ml/min/1.73m^2^	0 (0)	4 (20)	
Plasma potassium (mmol/L)	4.0 (3.9–4.3)	4.0 (3.6–4.1)	0.13
Plasma bicarbonate (mmol/L)	24.0 (23.0–25.0)	25.5 (24.0–27.8)	0.13
Plasma calcium (mmol/L)	2.32 (2.26–2.38)	2.39 (2.34–2.47)	0.02
Plasma phosphate (mmol/l)	1.08 (1.00—1.18)	1.05 (0.96–1.16)	0.60
Plasma magnesium (mmol/L)	0.79 (0.76–0.83)	0.82 (0.76–0.86)	0.47
Plasma uric acid (µmol/L)	299 (214–347)	323 (267–346)	0.32
PTH (ng/L)	44.0 (33.3–59.0)	40.5 (18.0–53.5)	0.54
25-hydroxyvitamin D (nmol/L)	72 (59–83)	79 (67–97)	0.15
1.25-dihydroxyvitamine D (pmol/L)	133 (114–148)	119 (91–141)	0.31
Urine volume (mL)	1900 (1500–2450)	2525 (2050–2963)	0.09
Creatinine tubular secretion (mL/min/1.73m^2^)	21.44 (− 8.1–34.0) (*n* = 11)	21.41 (2.9–31.7)	0 .80
Urine urea (mmol/24H)	335 (295–378)	328 (232–381)	0.55
Urine sodium (mmol/24H)	124 (87–154)	98 (65–134)	0.27
Urine potassium (mmol/24H)	55 (48–63)	69 (62–88)	0.02
Urine phosphate (mmol/24H)	19.6 (16.8–25.2)	20.2 (17.7–23.8)	0.91
Urine citrate (mmol/L)	1.53 (1.17–2.46)	0.88 (0.52–1.29)	0.002
Urine citrate (mmol/24H)	3.68 (2.70–5.62)	1.96 (1.12–3.39)	0.01
Urine calcium (mmol/L)	1.76 (1.41–2.70)	1.75 (1.13–2.37)	0.82
Urine calcium (mmol/kg/24H)	0.07 (0.05–0.09)	0.06 (0.05–0.08)	0.96
Urine oxalate (mmol/L)	0.13 (0.07–0.16)	0.10 (0.07–0.15)	> 0.99
Urine oxalate (mmol/24H)	0.26 (0.19–0.31)	0.25 (0.21–0.30)	0.75
Urine uric acid (mmol/L)	1.17 (0.85–1.8)	1.03 (0.89–1.17)	0.53
Urine uric acid (mmol/24H)	2.78 (2.24–3.12)	2.56 (2.42–2.94)	0.84
Proteinuria (g/24H)	0.11 (0.07–0.15)	0.18 (0.08–0.19)	0.03
Hematuria (RBC/mm^3^)	4 (1–10)	6 (4–10)	0.28
Leukocyturia (WBC/mm^3^)	2 (1–3)	11 (6–30)	< 0.0001
Urine pH	5.8 (5.4–6.2)	6.7 (6.2–7.1)	0.002
Urine density	1.014 (1.009–1.023)	1.015 (1.011–1.017)	0.84
Positive crystalluria	0 (0)	4 (20)	
Weddellite		1 (5)	
Whewellite + Weddellite		1 (5)	
Amorphous phosphate crystals		2 (10)	
Last renal colic (years)	NA	2.5 (1–5)	
Last pyelonephritis (years)	NA	2 (0–8)	
Last urologic intervention (years)	NA	1.5 (0–3)	
Age at first kidney stone (years)	NA	36 (22–42)	
Potassium citrate therapy	0 (0)	14 (70)	
Weight-adjusted citrate dose (mg/kg)	NA	41 (0–68)	
Thiazide diuretic therapy	0 (0)	8 (40)	
CKD Stage 1 (mGFR ≥ 90 ml/min/1.73m^2^)	8 (62)	2 (10)	
CKD Stage 2 (mGFR 60-89 ml/min/1.73m^2^)	5 (38)	14 (70)	
CKD Stage 3a (mGFR 45-59 ml/min/1.73m^2^)	0 (0)	4 (20)	
Hypocitraturia	0 (0)	12 (60)	
Hypercalciuria	1 (8)	2 (10)	
Hyperoxaluria	0 (0)	0 (0)	
Hypophosphatemia	0 (0)	1 (5)	
Hypokalemia	0 (0)	5 (25)	
Stone composition	NA		
Whewellite		4 (20)	
Carbapatite + Weddellite		3(20)	
Carbapatite		2 (10)	
Carbapatite + Whewellite		1 (5)	
Carbapatite + Whewellite + Weddellite		1 (5)	
Carbapatite + Brushite + Whewellite		1 (5)	
Carbapatite + Struvite		1 (5)	
Unknown		7 (35)	

^a^For quantitative variables, values are expressed as median (interquartile ranges). For qualitative variables, values are expressed as *n* (%). *NA* not applicable, *mGFR* measured glomerular filtration rate, *eGFR* estimated glomerular filtration rate, *PTH* Parathyroid hormone, *CKD* Chronic Kidney disease. Hypocitraturia was defined as urine citrate < 1.5 mmol/24H; hypokalemia was defined as kalemia < 3.5 mmol/L; hypophosphatemia was defined as phosphoremia < 0.81 mmol/L; hyperoxaluria was defined as urine oxalate > 0.5 mmol/24H; hypercalciuria was defined as urine calcium > 0.1 mmol/kg/24H and > 6.25 mmol/24 h for women and > 7.5 mmol/24 h for men

### Measured GFR

While eGFR did not differ between groups, mGFR was significantly lower in patients with medullary sponge kidney than in controls (78 ml/min/1.73m^2^ vs 90 ml/min/1.73m^2^; *p* = 0.008). CKD stage 3a was present in 4 (20%) patients with medullary sponge kidney but in none of the subjects in the control group. mGFR was significantly lower than eGFR only in the medullary sponge kidney group (78 ml/min/1.73m^2^ vs 91 ml/min/1.73m^2^; *p* < 0.0001) (Table 1). A difference of more than 5 ml/min/1.73m^2^ between mGFR and eGFR was observed in 16 medullary sponge kidney patients (Supplementary Material, table S2). We found no difference in tubular creatinine secretion between groups (*p* = 0.8).

### Morphological description

Cysts involving the medulla or corticomedullary junction were detected by MRI in 12 (60%) patients with medullary sponge kidney and in 0 (0%) controls. Median total kidney volume indexed to BSA (Controls: 98cm^3^/m^2^ (IQR 93–110) vs medullary sponge kidney: 94cm^3^/m^2^ (IQR 81–110), *p* = 0.4) and relative medullary volume (Controls: 0.43 cm^3^/ cm^3^ (IQR 0.36–0.48) vs medullary sponge kidney: 0.43cm^3^/ cm^3^ (0.35–0.48), *p* = 0.4) did not differ between groups (Table 2). No association was found between cyst presence and kidney volume. Relative medullary volume tended to be higher in patients with cysts than in those without (0.44 vs 0.36; *p* = 0.06).

**Table 2:** Morphological description and comparison of BOLD-MRI, DWI and T1 mapping -derived parameters in MSK and control group

Variables^a^	Controls (*n* = 13)	MSK (*n* = 20)	*p* value^1^
Time between MRI and the last CT scan (months)	2 (2–3)	8 (4–12)	
Renal cysts*	0 (0%)	12 (60%)	< 0.001
Number of cysts	NA	5 (3–8)	
Average cyst diameter (cm)	NA	1.25 (0.78–1.69)	
Kidney volume (cm^3^)	171 (144–211)	157 (141–197)	0.43
Kidney volume indexed to body surface area (cm^3^/m^2^)	98 (93–110)	94 (81–110)	0.35
Relative medullary volume	0.43 (0.36–0.48)	0.43 (0.35–0.48)	0.43
R2*			
Cortex	18.67 (17.22–19.68)	19.79 (18.30–21.83)	0.08
Medulla	34.14 (31.86–36.39)	33.23 (30.94–37.61)	0.67
Cortex-to-medulla ratio	0.55 (0.52–0.59)	0.60 (0.56–0.62)	0.04
ΔR2*	– 15.43 (– 16.89 to – 13.37)	– 13.84 (– 16.17 to – 12.06)	0.22
ADC			
Cortex	2.07 (1.99–2.15)	2.13 (2.06–2.23)	0.18
Medulla	1.74 (1.63–1.78)	1.75 (1.67–1.81)	0.48
Cortex-to-medulla ratio	1.22 (1.16–1.24)	1.23 (1.20–1.25)	0.55
ΔADC	0.37 (0.28–0.43)	0.40 (0.33–0.42)	0.46
T1			
Cortex	1430 (1401–1496)	1430 (1403–1476)	0.93
Medulla	1895 (1848–1943)	1850 (1808–1876)	0.048
Cortex-to-medulla ratio	0.75 (0.74–0.77)	0.78 (0.75–0.80)	0.11
ΔT1	– 480 (– 511 to – 405)	– 398 (– 456to – 357)	0.048

^**a**^For quantitative variables, values are expressed as median (interquartile ranges). For qualitative variables, values are expressed as *n* (%). *Renal cysts involving medulla or corticomedullary junction. ^1^ Comparison control vs MSK, Mann–Whitney U-test. R2* in s^−1^; ADC in 10^–3^ mm^2^/s; T1 in ms. *MSK* medullary sponge kidney, *ADC* apparent diffusion coefficient; Δ, corticomedullary difference

### fMRI findings in medullary sponge kidney patients and controls

A representative set of each fMRI sequence performed in a control and medullary sponge kidney patient is presented in Supplementary Material (Fig. S2).

#### Blood oxygen level-dependent imaging

**Fig. 1:**
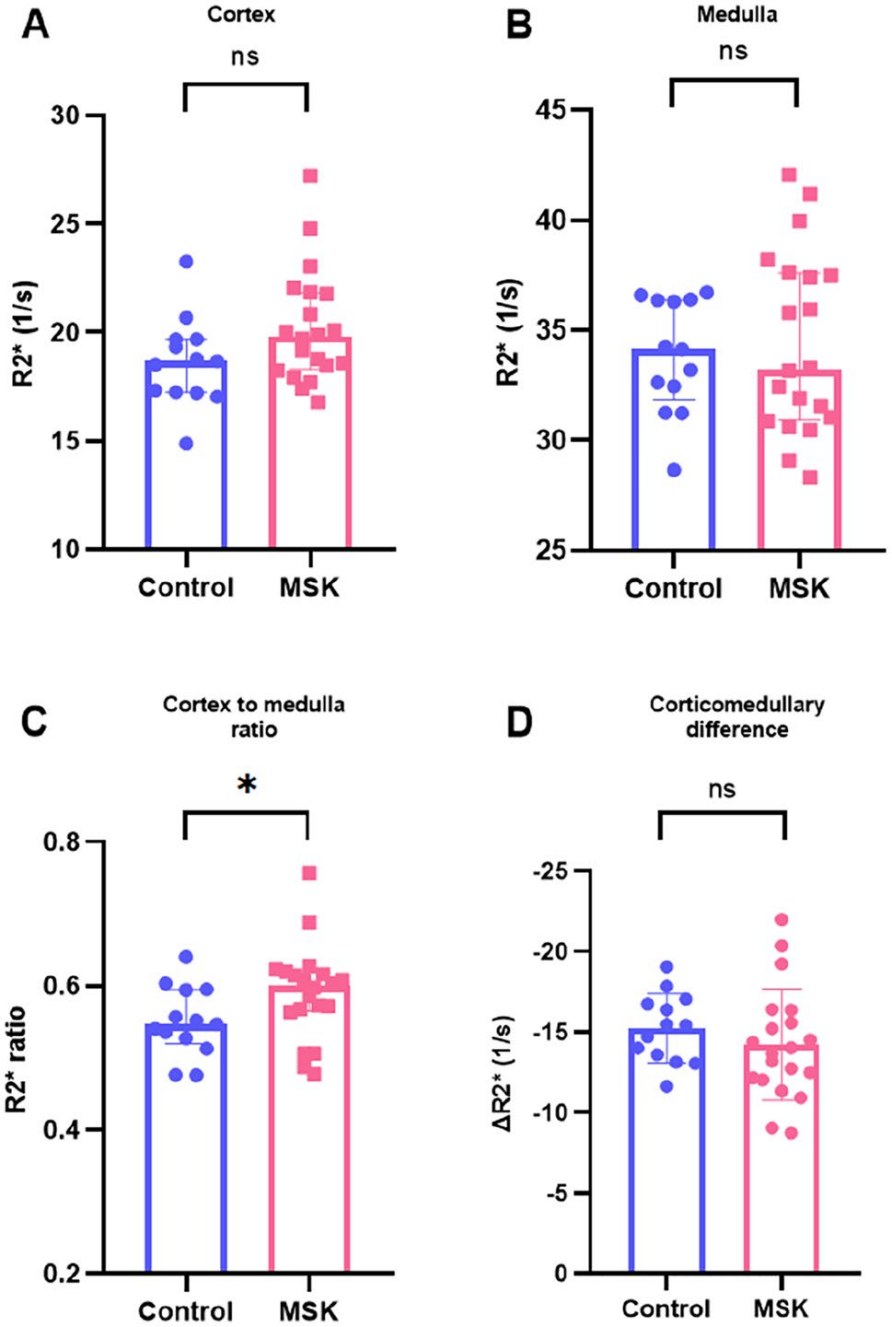
Comparison of Blood-Oxygen-Level-Dependent (BOLD)-derived R2 ∗ values between Medullary Sponge Kidney (MSK) patients and controls. **A** cortical R2* values, **B** medullary R2* values; **C** cortex-to-medulla ratio; **D** corticomedullary difference. R2* in s^−1^. *MSK* medullary sponge kidney; *, *p* < 0.05; *ns* not significant. Comparison control vs MSK, Mann–Whitney U-test

Cortical R2* was lower than medullary R2* in both groups (Controls: 18.67 s^−1^ vs 34.14 s^−1^; *p *< 0.001; medullary sponge kidney: 19.79 s^−1^ vs 33.23 s^−1^; *p* < 0.001) (Table 2**)**. Cortical R2* tended to be higher in patients with medullary sponge kidney compared to controls (19.79 s^−1^ vs 18.67 s^−1^; *p* = 0.08) (Fig. 1A). Medullary R2* was not different between groups (Fig. 1B). The R2* cortex-to-medulla ratio was significantly higher in medullary sponge kidney patients than in controls (0.60 vs 0.55; *p* = 0.04) (Fig. 1C). The corticomedullary R2* difference was not different between groups (Fig. 1D).

#### Diffusion-weighted imaging

**Fig. 2:**
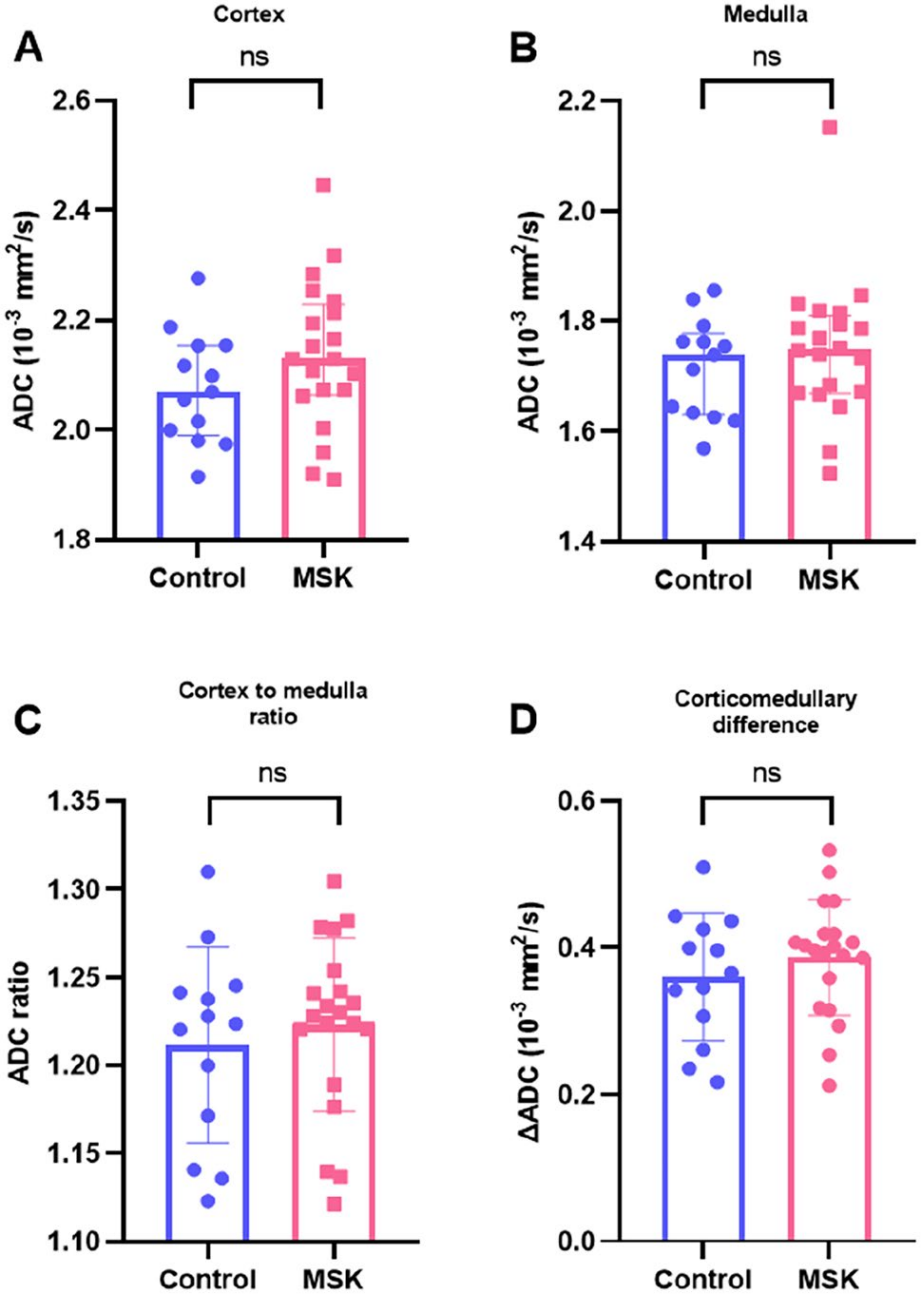
Comparison of Diffusion-Weighted Imaging (DWI)-derived Apparent Diffusion Coefficient (ADC) values between Medullary Sponge Kidney (MSK) patients and controls. **A** cortical ADC values, **B** medullary ADC values; **C** cortex-to-medulla ratio; **D** corticomedullary difference. ADC in 10^–3^ mm^2^/s. *MSK* medullary sponge kidney, *ADC* Apparent diffusion coefficient, *ns* not significant. Comparison control vs MSK, Mann–Whitney U-test

Cortical apparent diffusion coefficient was higher than medullary apparent diffusion coefficient in both the control (2.07*10^−3^mm^2^/s vs 1.74*10^−3^mm^2^/s; *p* < 0.001) and the medullary sponge kidney groups (2.13*10^−3^mm^2^/s vs 1.75*10^−3^mm^2^/s; *p* < 0.001) (Table 2). Cortical and medullary apparent diffusion coefficient (Fig. 2A and B) as well as apparent diffusion coefficient cortex-to-medulla ratio and corticomedullary apparent diffusion coefficient difference (Fig. 2C and D) were not different between groups.

#### T1 mapping

Cortical T1 was lower than medullary T1 in both groups (Controls: 1430 ms vs 1895 ms; *p* < 0.0001; medullary sponge kidney: 1430 ms vs 1850 ms; *p* < 0.001) (Table 2). Medullary T1 was significantly lower in medullary sponge kidney patients compared to controls (1850 ms vs 1895 ms; *p* = 0.048) (Supplementary Materials, Fig.S3B). Corticomedullary T1 difference (ΔT1) was significantly higher in controls compared to medullary sponge kidney patients (− 480 ms vs − 398 ms; *p* = 0.048). No difference between groups was observed for cortical T1 and T1 cortex-to-medulla ratio (Supplementary Materials Fig.S3A and C).

### Associations between MRI and clinical/biological data in medullary sponge kidney patients

Patients treated with potassium citrate had significantly higher cortical R2* compared to those who were untreated (20.06 s^−1^ vs 18.19 s^−1^; *p* = 0.03). Weight-adjusted citrate dose was significantly correlated with cortical R2* (r = 0.59; *p* = 0.006) and with medullary R2* (r = 0.46; p = 0.04), but not with R2* cortex-to-medulla ratio (Supplementary Materials, Fig.S4). No association between thiazide diuretic therapy and R2* was observed. No other correlation was found between R2* and biology. No association was noted between apparent diffusion coefficient and biology, including mGFR and eGFR. Medullary T1 correlated significantly with GFR (r = 0.50; *p* = 0.02 for mGFR and r = 0.55; *p* = 0.01 for eGFR). No other significant correlation was found between T1 values and biological parameters. Medullary sponge kidney severity according to the number of affected papillae on CT scan did not influence MRI results except for medullary T1 (Supplementary Materials, Table S3). The time between MRI and the last urological intervention, renal colic and episode of pyelonephritis did not influence MRI results.

## Discussion

This study aimed to provide a deep phenotyping of patients affected by medullary sponge kidney. We showed that the usual eGFR formula overestimates kidney function. We confirmed that morphological abnormalities with kidney cysts are detected with MRI. Furthermore, we  identified impaired renal oxygenation in medullary sponge kidneys. We found no evidence of kidney fibrosis. Hypocitraturia was the most frequent urinary abnormality found in medullary sponge kidney patients.

We highlighted that early kidney function impairment was common in medullary sponge kidney patientss, overestimating GFR by 11 mL/min/1.73m^2^. CKD stage 3a was found  in 20% of our patients by direct assessment. We did not find any difference in tubular creatinine secretion and in muscle mass. Indeed, CKD is considered uncommon in medullary sponge kidney, as it primarily affects patients with secondary struvite stones and/or pyelonephritis and contralateral congenital small kidneys [2]. However, in our study, only one patient had a history of struvite stones, and kidney volumes were similar between groups. Longitudinal studies are needed to assess GFR decline in medullary sponge kidney patients.

We confirmed that medullary sponge kidney is characterized by pre-calyceal dilatation of renal tubules, forming a cyst-like appearance. MRI revealed cysts located in the medulla/corticomedullary junction in 60% of patients, supporting the hypothesis of medullary microstructure alteration. We highlighted once again the heterogeneity of  medullary sponge kidney phenotype  and we found no difference in citraturia or urine pH between patients with medullary sponge kidney with and without cysts. This supports a link between medullary sponge kidney and ciliopathies, as suggested by the association with *PKHD1* variants. Indeed, Letavernier et al. identified biallelic *PKHD1* variants in two medullary sponge kidney patients, and heterozygous *PKHD1* mice developed tubular ectasia [16, 17]. However, we lacked complete genetic data in this cohort to test this hypothesis. Overall kidney size remained normal in these observations, consistent with our findings, whereas classical autosomal recessive polycystic kidney disease is associated with enlarged kidneys.

We observed significant corticomedullary impairment for renal oxygen content measured with blood-oxygen-level-dependent MRI in patients with medullary sponge kidney compared to controls. Corticomedullary gradient is a hallmark of tubular function and has been shown to reflect urine-concentrating ability using sodium MRI [18]. The reduced gradient detected via fMRI further supports the notion of tubular dysfunction in medullary sponge kidney [2]. Herein, R2* values did not correlate with GFR, consistent with other studies [19–22]. However, this reduced corticomedullary gradient observed on blood-oxygen-level-dependent MRI suggests impaired oxygenation as seen in CKD, where an imbalance in oxygen delivery and consumption contributes to hypoxia which is a key factor in the onset and progression of the disease and could explain this discrepancy in kidney function between groups [23, 24].

Cortical R2* was higher in patients treated with potassium citrate. Both cortical and medullary R2* correlated with weight-adjusted citrate dose. Hypocitraturia is a surrogate marker for collecting duct dysfunction, and potassium citrate therapy is the cornerstone of medical management for recurrent nephrolithiasis by preventing crystallization [4]. Therefore, our results suggest a correlation between medullary sponge kidney severity, i.e. higher stone rate justifying potassium citrate therapy, and kidney hypoxia. We showed that thiazides did not modify the blood-oxygen-level-dependent signal, contrary to loop diuretics, and might not be associated with medullary sponge kidney severity [6]. Increased R2* has already been identified as a predictor of kidney function decline, independently of other known predictors of CKD progression [25, 26]. Thus, cortical R2* could serve as a tool for identifying the most severe medullary sponge kidney patients. Nevertheless, these assumptions remain speculative and need to be confirmed.

We found no difference in apparent diffusion coefficient. The primary potential of renal diffusion-weighted imaging MRI lies in its non-invasive assessment of interstitial fibrosis [27]. The lack of difference in apparent diffusion coefficient in our cohort aligns with the preserved cortical structure and the absence of interstitial fibrosis in medullary sponge kidney, as described by Ekstrom et al. and Evan et al. [28, 29]. The only case report of renal MRI in medullary sponge kidney reported low apparent diffusion coefficient (1.12*10^–3^ mm^2^/s) on the side of the papilla that may in fact reflect a blood clot in the cystic structures [9].

Although we observed a significant reduction in medullary T1 in medullary sponge kidney patients compared to controls, cortical T1 remained unchanged. Additionally, there was no significant difference in the cortex-to-medulla T1 ratio between groups. Among the few studies that have included histological analysis of native kidneys, most found an association between fibrosis and higher cortical T1 [21, 30]. As discussed above, no interstitial fibrosis was observed in the two medullary sponge kidney series with available histology, consistent with the absence of difference in cortical T1 between groups. Interestingly, one medullary sponge kidney patient in our study was an outlier according to T1 values (cortical T1: 1040 ms, medullary T1: 1467 ms). This patient was the only one who presented important microcalculi in dilated terminal tubules on CT scan. When this outlier was excluded from statistical analysis, our conclusions regarding blood-oxygen-level-dependent MRI remained consistent. Similar observations were described in cardiac fMRI, with decreased T1 on caseous calcification of the mitral annulus and myocardial calcification [31, 32]. This suggests that medullary T1 modifications detected in medullary sponge kidney patients might in fact be due to calcifications. These preliminary observations require confirmation in larger studies.

Our study has limitations. The number of participants available for analysis was limited with only 13 controls included out of the 20 initially planned, due to installation, during the inclusion period, of a new MRI-scanner to replace the 3.0-T MRI-scanner used for the study. Controls were unmatched adults without history of kidney stones. We had more women in the medullary sponge kidney group, however no sex difference has been reported in the literature for fMRI and we found none in our cohort. Blood-oxygen-level-dependent signal is influenced by diet, however it was probably negligible as 24-h urinary volumes and natriuresis were comparable between groups, and the study protocol was standardized as much as possible [6]. Each image was measured independently by four readers to prevent significant bias from measurement variability and to consider the wider cortical and medullary area since a blood-oxygen-level-dependent semi-automated analysis method was not available in our center (Supplementary Materials, table S4) [6]. We did not include outer medulla region of interest selection becauseit was hard to locate and a high interoperator variability has been reported in this layer [6].

## Conclusion

We are herein reporting deep phenotyping of a series of patients with medullary sponge kidney, providing interesting new data: first that GFR is overestimated and CKD is not rare in patients affected by medullary sponge kidney, secondly, MRI can visualize medullary cystic appearance of medullary sponge kidney, and finally, we identified impaired renal oxygenation in medullary sponge kidney on blood-oxygen-level-dependent MRI. Further research is needed, including longitudinal studies, to better understand the prognosis and evaluate the slope of GFR decline in this disease.

## Supplementary Information

Below is the link to the electronic supplementary material.

Supplementary file1 (DOCX 4906 KB)

## Data Availability

We will send raw data on request. Data described in the manuscript will be made available upon request pending approval.

## References

[1] Baert L (1978) Microdissection findings of medullary sponge kidney. Urology Juin 11(6):637–640 10.1016/0090-4295(78)90021-3 675935

[2] Fabris A, Anglani F, Lupo A, Gambaro G (2013) Medullary sponge kidney: state of the art. Nephrol Dialy Transplant 28(5):1111–1119 10.1093/ndt/gfs505 23229933

[3] McPhail EF, Gettman MT, Patterson DE, Rangel LJ, Krambeck AE (2012) Nephrolithiasis in medullary sponge kidney: evaluation of clinical and metabolic features. Urology févr 79(2):277–281 10.1016/j.urology.2011.07.1414 22014971

[4] Fabris A, Lupo A, Bernich P, Abaterusso C, Marchionna N, Nouvenne A et al (2010) Long-term treatment with potassium citrate and renal stones in medullary sponge kidney. Clin J Am Soc Nephrol 5(9):1663–1668 20576821 10.2215/CJN.00220110 PMC2974409

[5] Tournebize C, Schleef M, De Mul A, Pacaud S, Derain-Dubourg L, Juillard L et al. 2025. Multiparametric MRI: can we assess renal function differently. Clin Kidn J. 18(1):sfae365. 10.1093/ckj/sfae365 PMC11852294 40008350

[6] Pruijm M, Mendichovszky IA, Liss P, Van Der Niepen P, Textor SC, Lerman LO et al (2018) Renal blood oxygenation level-dependent magnetic resonance imaging to measure renal tissue oxygenation: a statement paper and systematic review. Nephrol Dialy Transplant 33(2):22–28 10.1093/ndt/gfy243 PMC6106642 30137579

[7] Caroli A, Schneider M, Friedli I, Ljimani A, De Seigneux S, Boor P et al (2018) Diffusion-weighted magnetic resonance imaging to assess diffuse renal pathology: a systematic review and statement paper. Nephrol Dial Transplant 33(2):29–40 10.1093/ndt/gfy163 PMC6106641 30137580

[8] Wolf M, De Boer A, Sharma K, Boor P, Leiner T, Sunder-Plassmann G et al (2018) Magnetic resonance imaging T1- and T2-mapping to assess renal structure and function: a systematic review and statement paper. Nephrol Dial Transplant 33(2):41–50 10.1093/ndt/gfy198 PMC6106643 30137583

[9] Hida T, Nishie A, Asayama Y, Ishigami K, Fujita N, Inokuchi J et al (2012) MR imaging of focal medullary sponge kidney: case report. MRMS 11(1):65–69 22450389 10.2463/mrms.11.65

[10] Gaunay GS, Berkenblit RG, Tabib CH, Blitstein JR, Patel M, Hoenig DM (2018) Efficacy of multi-detector computed tomography for the diagnosis of medullary sponge kidney. Curr Urol Mar 11(3):139–143 10.1159/000447208 PMC5903462 29692693

[11] Almeras C, Daudon M, Ploussard G, Gautier JR, Traxer O, Meria P (2016) Endoscopic description of renal papillary abnormalities in stone disease by flexible ureteroscopy: a proposed classification of severity and type. World J Urol 34(11):1575–1582 27033084 10.1007/s00345-016-1814-6

[12] Du Bois D, Du Bois EF (1989) A formula to estimate the approximate surface area if height and weight be known. 1916. Nutrition 5(5):303–311 2520314

[13] Levey AS, Stevens LA, Schmid CH, Zhang Y et al (2009) A new equation to estimate glomerular filtration rate. Ann Intern Med 150(9):604 19414839 10.7326/0003-4819-150-9-200905050-00006 PMC2763564

[14] Walser M (1987) Creatinine excretion as a measure of protein nutrition in adults of varying age. JPEN J Parenter Enteral Nutr 11(5 Suppl):73S-78S 3312696 10.1177/014860718701100510

[15] Ix JH, Wassel CL, Stevens LA, Beck GJ, Froissart M, Navis G et al (2011) Equations to estimate creatinine excretion rate: the CKD epidemiology collaboration. Clin J Am Soc Nephrol 6(1):184–191 (**janv**) 20966119 10.2215/CJN.05030610 PMC3022241

[16] Letavernier E, Schwoehrer M, Livrozet M, Saint-Jacques C, Raymond L, Saraeva R et al (2022) Atypical clinical presentation of autosomal recessive polycystic kidney mimicking medullary sponge kidney disease. Kidney Intern Report 7(4):916–919 10.1016/j.ekir.2021.11.035 PMC9039475 35497799

[17] Shan D, Rezonzew G, Mullen S, Roye R, Zhou J, Chumley P et al (2019) Heterozygous *Pkhd1*^C642*^ mice develop cystic liver disease and proximal tubule ectasia that mimics radiographic signs of medullary sponge kidney. Am J Physiol-Renal Physiol 316(3):463–472 10.1152/ajprenal.00181.2018 PMC6442377 30600684

[18] Akbari A, Lemoine S, Salerno F, Marcus TL, Duffy T, Scholl TJ et al (2022) Functional sodium MRI helps to measure corticomedullary sodium content in normal and diseased human kidneys. Radiology 303(2):384–389 35133199 10.1148/radiol.211238

[19] Michaely HJ, Metzger L, Haneder S, Hansmann J, Schoenberg SO, Attenberger UI (2012) Renal BOLD-MRI does not reflect renal function in chronic kidney disease. Kidney Intern 81(7):684–689 10.1038/ki.2011.455 22237750

[20] Pruijm M, Hofmann L, Piskunowicz M, Muller ME, Zweiacker C, Bassi I, et al. Determinants of Renal Tissue Oxygenation as Measured with BOLD-MRI in Chronic Kidney Disease and Hypertension in Humans. Joles JA, éditeur. PLoS ONE. 2014. 9(4):e95895. 10.1371/journal.pone.0095895 PMC3997480 24760031

[21] Buchanan CE, Mahmoud H, Cox EF, McCulloch T, Prestwich BL, Taal MW et al (2020) Quantitative assessment of renal structural and functional changes in chronic kidney disease using multi-parametric magnetic resonance imaging. Nephrol Dial Transplant 35(6):955–964 31257440 10.1093/ndt/gfz129 PMC7282828

[22] Evans RG, Leong CL, Anderson WP, O’Connor PM (2007) Don’t be so BOLD: potential limitations in the use of BOLD MRI for studies of renal oxygenation. Kid Intern 71(12):1327–1328 10.1038/sj.ki.5002321 17554358

[23] Li C, Liu H, Li X, Zhou L, Wang R, Zhang Y (2019) Application of BOLD-MRI in the classification of renal function in chronic kidney disease. Abdom Radiol 44(2):604–611 (**févr**) 10.1007/s00261-018-1750-6 30151714

[24] Fine LG, Norman JT (2008) Chronic hypoxia as a mechanism of progression of chronic kidney diseases: from hypothesis to novel therapeutics. Kidney Int 74(7):867–872 18633339 10.1038/ki.2008.350

[25] Pruijm M, Milani B, Pivin E, Podhajska A, Vogt B, Stuber M et al (2018) Reduced cortical oxygenation predicts a progressive decline of renal function in patients with chronic kidney disease. Kidney Int 93(4):932–940 29325997 10.1016/j.kint.2017.10.020

[26] Sugiyama K, Inoue T, Kozawa E, Ishikawa M, Shimada A, Kobayashi N et al (2020) Reduced oxygenation but not fibrosis defined by functional magnetic resonance imaging predicts the long-term progression of chronic kidney disease. Nephrol Dial Transplant 35(6):964–970 30418615 10.1093/ndt/gfy324

[27] Friedli I, Crowe LA, Berchtold L, Moll S, Hadaya K, De Perrot T et al (2016) New magnetic resonance imaging index for renal fibrosis assessment: a comparison between diffusion-weighted imaging and T1 mapping with histological validation. Sci Rep 6(1):30088 27439482 10.1038/srep30088 PMC4954968

[28] Medullary sponge kidney. By Tore Ekström, Bengt Engfeldt, Curt Lagergren, and Nils Lindvall. 9½ × 6¼ in. Pp. 73, with 28 pages of illustrations. 1960. Stockholm: Almquist and Wiksell. Sw.Kr. 18. British Journal of Surgery. 6 déc 2005;48(211):583–583

[29] Evan AP, Worcester EM, Williams JC, Sommer AJ, Lingeman JE, Phillips CL et al (2015) Biopsy proven medullary sponge kidney: clinical findings, histopathology, and role of osteogenesis in stone and plaque formation. Anatom Record 298(5):865–877 10.1002/ar.23105 PMC4405475 25615853

[30] Mao W, Ding X, Ding Y, Cao B, Fu C, Kuehn B et al (2022) Evaluation of interstitial fibrosis in chronic kidney disease by multiparametric functional MRI and histopathologic analysis. Eur Radiol 33(6):4138–4147 36502460 10.1007/s00330-022-09329-7

[31] Tanaka Y, Hamatani Y, Iguchi M, Minami K, Ishigami K, Ikeda S et al (2022) Caseous calcification of mitral annulus evaluated by multi-modality imaging including cardiac magnetic resonance parametric mapping. J Cardiol Cases 26(3):221–224 36091606 10.1016/j.jccase.2022.04.019 PMC9449737

[32] Nijjar PS, Okasha O (2019) Multiparametric cardiovascular magnetic resonance imaging of acute myocardial calcification. Europ Heart J 20(3):371–371 10.1093/ehjci/jey204 30561587

