# Supplementary material for: Medullary sponge kidney: in-depth phenotyping for a better understanding of functional and structural abnormalities
Source: J Nephrol. 2025 Sep 19;38(8):2363–73. doi: 10.1007/s40620-025-02413-3 (PMC12630228; doi:10.1007/s40620-025-02413-3)
Supplement: Supplementary file 1 — Supplementary file1 (DOCX 4906 KB) [file 40620_2025_2413_MOESM1_ESM.docx]

Supplementary Materials

***Table S1 MRI parameters***

|  | **T2** | **BOLD** | **DWI** | **T1 MAPPING** |
| --- | --- | --- | --- | --- |
| **Orientation** | Coronal | Coronal | Coronal | Coronal |
| **Voxel volume (mm)** | 0.95 x 1.1 x 4 | 1.9 x 1.9 x 6 | 1.8 x 1.8 x 3 | 3 x 3.03 x 6 |
| **Repetition time (ms)** | 1200 | 1.2 | 3000 | 2.5 |
| **Echo time (ms)** | 80 | 80 | 70 | 1.2 |
| **Matrix** | 412 x 354 | 184 x 145 | 156 x 67 | 112 x 98 |
| **Number of slices** | 36 | 1 | 3 | 1 |
| **Field of vue** | 390 x 390 | 345 x 276 | 280 x 120 | 330 x 296 |
| **Flip angle (°)** | 90 | 30 | 90 | 20 |
| **Acquisition time (s)** | 44 | 18 | 135 | 11 |
| **b value** |  |  | 0, 800 |  |

***Table S2* difference between eGFR and mGFR for MSK patients**

| eGFR (ml/min/1.73m²) | mGFR  (ml/min/1.73m²) | mGFR – eGFR  (ml/min/1.73m²) |
| --- | --- | --- |
| 90 | 85 | -5 |
| 73 | 57 | -16 |
| 72 | 75 | 3 |
| 66 | 69 | 3 |
| 105 | 85 | -20 |
| 78 | 63 | -15 |
| 106 | 99 | -7 |
| 66 | 55 | -11 |
| 74 | 51 | -23 |
| 106 | 78 | -28 |
| 85 | 78 | -7 |
| 84 | 72 | -12 |
| 64 | 59 | -5 |
| 105 | 83 | -22 |
| 106 | 82 | -24 |
| 102 | 91 | -11 |
| 99 | 81 | -18 |
| 98 | 87 | -11 |
| 100 | 73 | -27 |
| 92 | 79 | -13 |

mGFR, measured glomerular filtration rate; eGFR, estimated glomerular filtration rate (CKDEPI)

***Table S3 MRI parameters according to MSK severity on CT scan***

| **MSK severity**  **Variables** | **Bilateral and panrenal**  **n = 16** | **Unilateral**  **n = 1** | **Bilateral and involving not all the papillae**  **n = 3** | **p** |
| --- | --- | --- | --- | --- |
| **R2*** |  |  |  |  |
| Cortex | 19.8 (18.3 – 21.6) | 17.7 | 20.3 (18.0 – 22.7) | 0.3 |
| Medulla | 32.8 (30.7 – 38.1) | 30.9 | 35.8 (33.3 – 37.4) | 0.6 |
| Cortex-to-medulla ratio | 0.60 (0.52 – 0.62) | 0.57 | 0.61 (0.56 – 0.62) | 0.7 |
| ΔR2* | -13.2 (-16.4 - -11.5) | -13.2 | -14.4 (-14.5 - -14.0) | 0.3 |
| **ADC** |  |  |  |  |
| Cortex | 2.12 (2.02 – 2.23) | 2.20 | 2.17 (2.06 – 2.45) | 0.9 |
| Medulla | 1.74 (1.67 – 1.81) | 1.79 | 1.77 (1.67 – 2.15) | 0.8 |
| Cortex-to-medulla ratio | 1.23 (1.20 – 1.27) | 1.23 | 1.22 (1.14 – 1.24) | 0.8 |
| ΔADC | 0.40 (0.33 – 0.45) | 0.41 | 0.39 (0.29 – 0.40) | 0.6 |
| **T1** |  |  |  |  |
| Cortex | 1431 (1403 – 1494) | 1438 | 1418 (1367 – 1469) | 0.8 |
| Medulla | 1857 (1813 – 1876) | 1920 | 1765 (1763 – 1813) | 0.03 |
| Cortex-to-medulla ratio | 0.78 (0.75 – 0.79) | 0.75 | 0.80 (0.78 – 0.81) | 0.4 |
| ΔT1 | -411 (-456 - -387) | -482 | -347 (-396 - -343) | 0.9 |

**V**alues are expressed as median (interquartile ranges). ^1^ Comparison between the groups, Kruskal-Wallis test. Values are expressed as median (interquartile range); R2* in s^-1^; ADC in 10^-3^ mm²/s; T1 in ms. MSK, medullary sponge kidney; ADC, apparent diffusion coefficient; Δ, corticomedullary difference

**Table S4 Coefficient of variation of each operator**

| **Operator**  **Sequence** | **SL** | **OR** | **SP** | **CT** |
| --- | --- | --- | --- | --- |
| **BOLD** |  |  |  |  |
| **Cortex** | 14.56% | 14.40% | 13.67% | 14.95% |
| **Medulla** | 12.65% | 17.79% | 12.81% | 13.25% |
| **DWI** |  |  |  |  |
| **Cortex** | 6.27% | 6.33% | 7.77% | 7.05% |
| **Medulla** | 8.3% | 7.92% | 6.45% | 7.63% |
| **T1 MAPPING** |  |  |  |  |
| **Cortex** | 6.36% | 6.75% | 5.96% | 7.02% |
| **Medulla** | 6.99% | 6.09% | 5.47% | 6.92% |

**
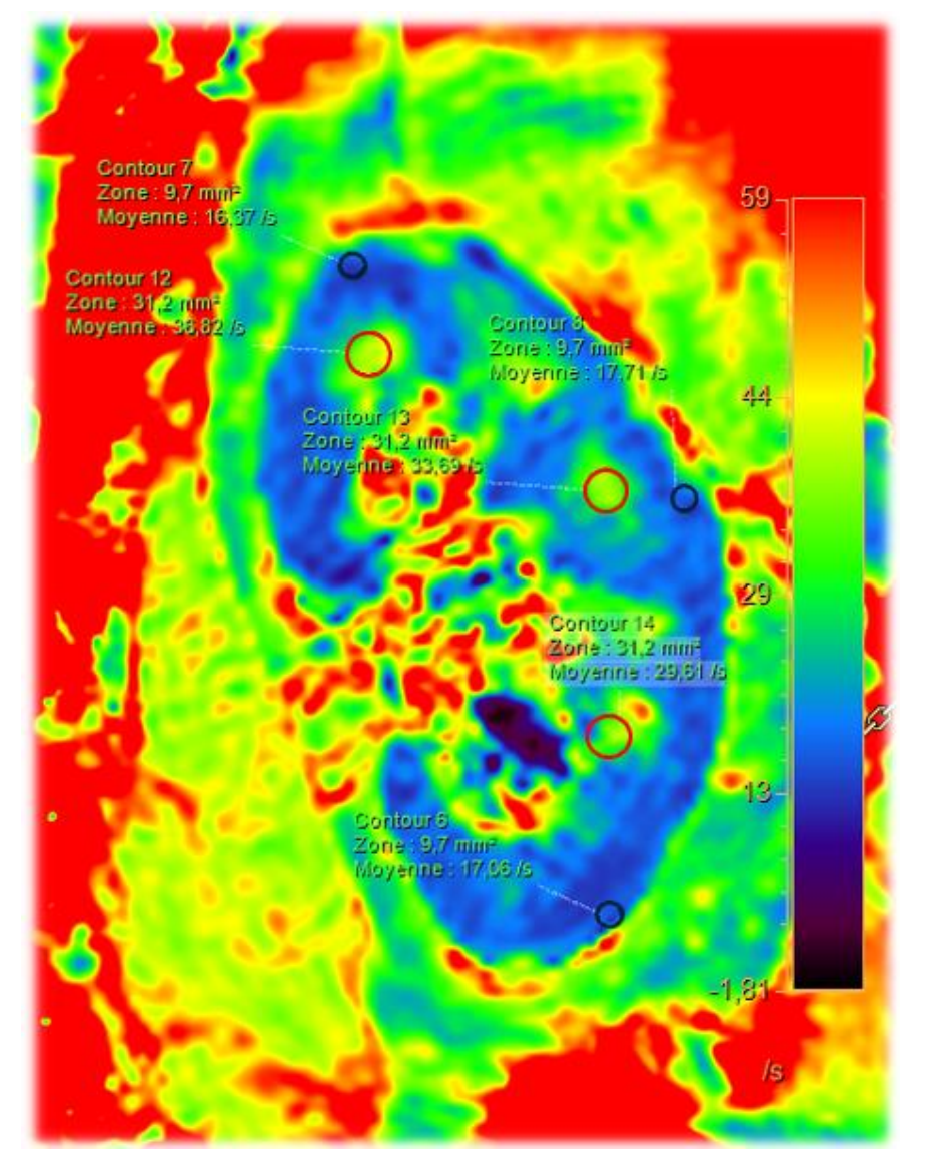
**

**Figure S1: Representative example of a left kidney BOLD MRI imaging with ROI selection.**

Red circles = medulla; black circles = cortex. BOLD MRI, Blood oxygen level dependent magnetic resonance imaging; ROI, Region of interest

**
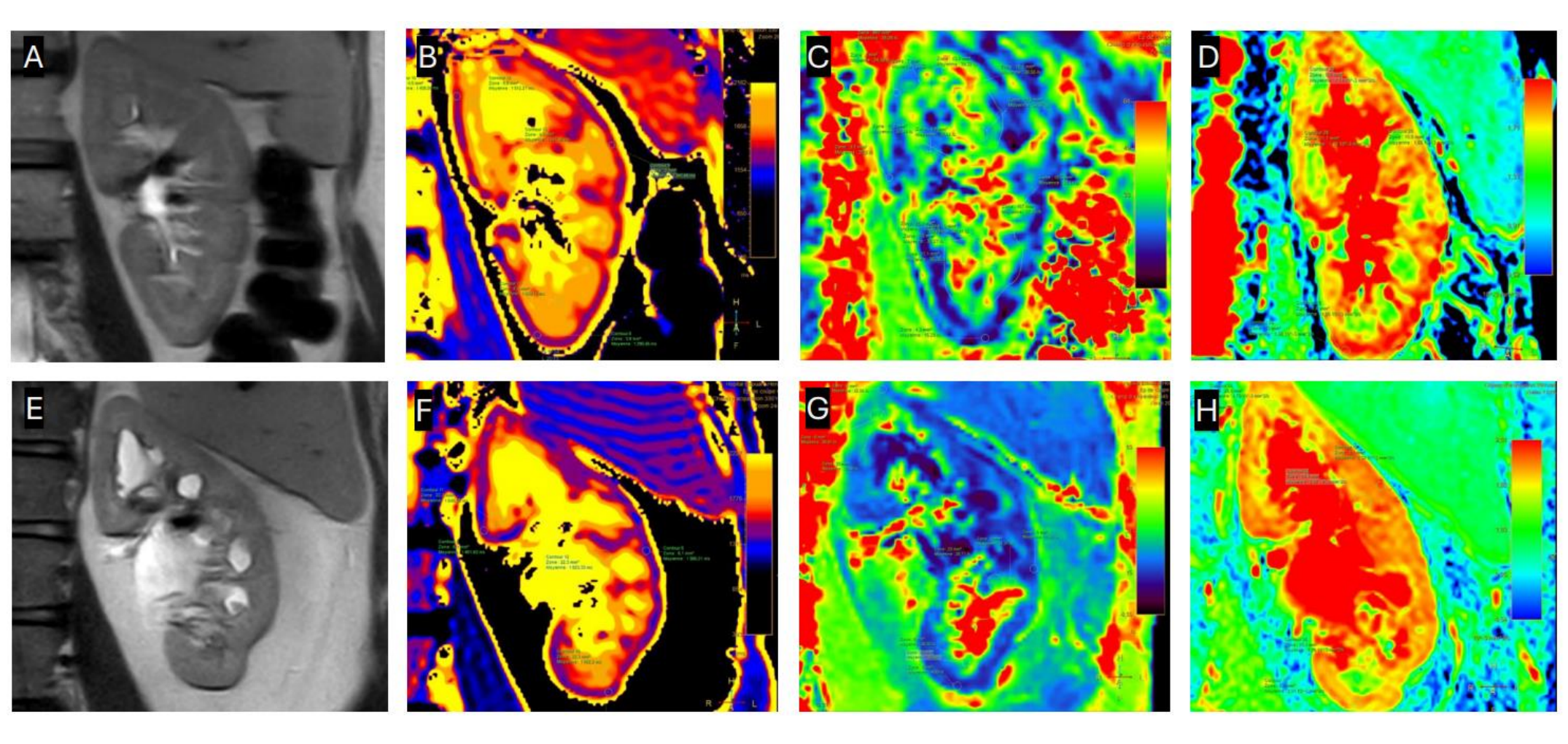
**

**Figure S2:** **Representative set of a left kidney multiparametric MRI (coronal plane) imaging in a control and a MSK patient**

Upper row: Control participant; lower row: MSK patient. (A, E) T2 morphological study; (B, F) colored T1 mapping images; (C, G) colored BOLD images; (D, H) colored DWI images.

MSK, medullary sponge kidney; BOLD, Blood oxygen level dependent; DWI, diffusion weighted imaging.


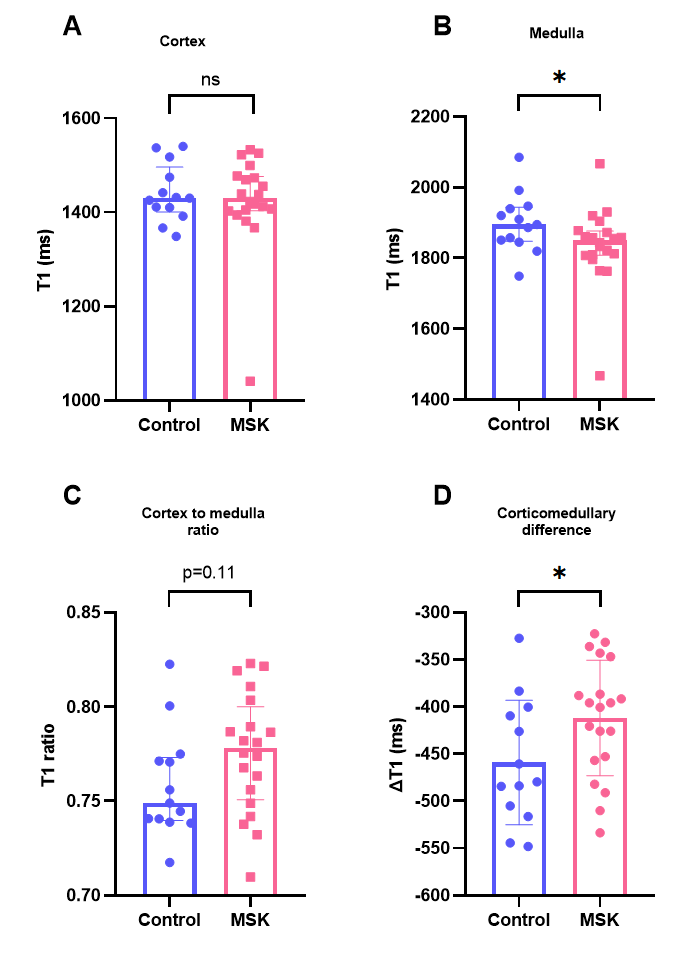


**Fig. S3: Comparison of T1-mapping derived T1 relaxation times values between MSK and control.** (A) cortical T1 values, (B) medullary T1 values; (C) cortex to medulla ratio; (D) corticomedullary difference. T1 in ms. MSK, medullary sponge kidney. *, p < 0.05; ns, not significant. Comparison control vs MSK, Mann–Whitney U-test


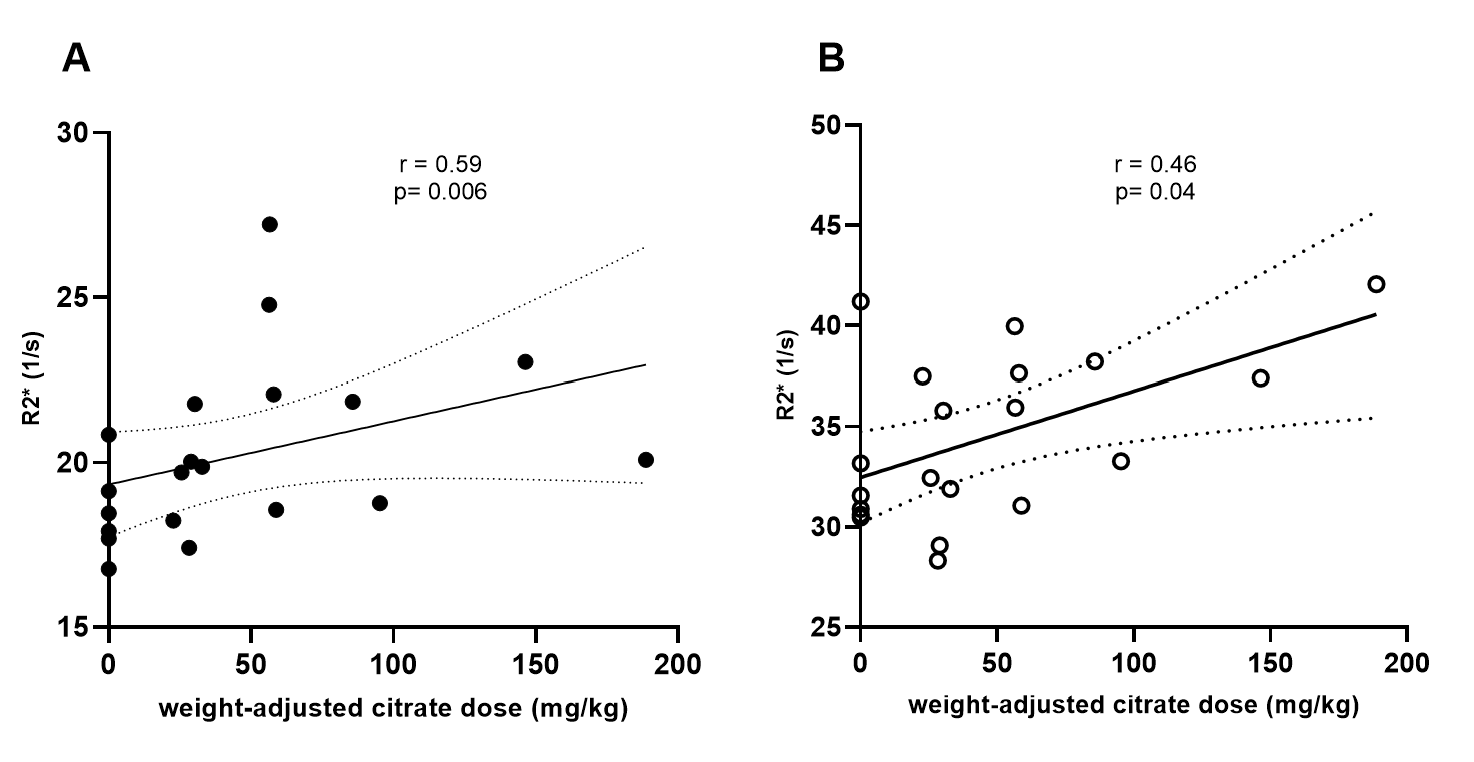


**Fig. S4: Correlation between R2* values and weighted-adjusted citrate dose in MSK patients.** Correlation between cortical (A) and medullary (B) R2* values and weighted-adjusted citrate dose in MSK patients. Correlation tested by Spearman test. MSK, medullary sponge kidney
